# From wheat straw to bioethanol: integrative analysis of a separate hydrolysis and co-fermentation process with implemented enzyme production

**DOI:** 10.1186/s13068-015-0232-0

**Published:** 2015-03-18

**Authors:** Vera Novy, Karin Longus, Bernd Nidetzky

**Affiliations:** Institute of Biotechnology and Biochemical Engineering, Graz University of Technology, NAWI Graz, Petersgasse 12/I, 8010 Graz, Austria; Austrian Centre of Industrial Biotechnology, Petersgasse 14, A-8010 Graz, Austria

## Abstract

**Background:**

Lignocellulosic ethanol has a high potential as renewable energy source. In recent years, much research effort has been spent to optimize parameters involved in the production process. Despite that, there is still a lack of comprehensive studies on process integration. Single parameters and process configurations are, however, heavily interrelated and can affect the overall process efficiency in a multitude of ways. Here, we present an integrative approach for bioethanol production from wheat straw at a representative laboratory scale using a separate hydrolysis and co-fermentation (SHCF) process. The process does not rely on commercial (hemi-) cellulases but includes enzyme production through *Hypocrea jecorina* (formerly *Trichoderma reesei*) on the pre-treated feedstock as key unit operation. Hydrolysis reactions are run with high solid loadings of 15% dry mass pre-treated wheat straw (DM WS), and hydrolyzates are utilized without detoxification for mixed glucose-xylose fermentation with the genetically and evolutionary engineered *Saccharomyces cerevisiae* strain IBB10B05.

**Results:**

Process configurations of unit operations in the benchtop SHCF were varied and evaluated with respect to the overall process ethanol yield (*Y*_Ethanol-Process_). The highest *Y*_Ethanol-Process_ of 71.2 g ethanol per kg raw material was reached when fungal fermentations were run as batch, and the hydrolysis reaction was done with an enzyme loading of 30 filter paper units (FPU)/g_DM WS_. 1.7 ± 0.1 FPU/mL were produced, glucose and xylose were released with a conversion efficiency of 67% and 95%, respectively, and strain IBB10B05 showed an ethanol yield of 0.4 g/g_Glc + Xyl_ in 15% hydrolyzate fermentations. Based on the detailed process analysis, it was further possible to identify the enzyme yield, the glucose conversion efficiency, and the mass losses between the unit operations as key process parameters, exhibiting a major influence on *Y*_Ethanol-Process_.

**Conclusions:**

*Y*_Ethanol-Process_ is a measure for the efficiency of the lignocellulose-to-bioethanol process. Based on mass balance analysis, the correlations between single process parameters and *Y*_Ethanol-Process_ were elucidated. The optimized laboratory scale SHCF process showed efficiencies similar to pilot scale plants. The herein presented process analysis can serve as effective and simple tool to identify key process parameters, bottlenecks, and future optimization targets.

**Electronic supplementary material:**

The online version of this article (doi:10.1186/s13068-015-0232-0) contains supplementary material, which is available to authorized users.

## Background

Utilization of biomass as renewable and sustainable energy source has called attention from politics and R&D facilities around the world [1-5]. Second-generation bioethanol produced from lignocellulosic waste streams constitutes the most feasible technical option. Reasons are, among many, the geographically evenly distributed and inexpensive feedstock as well as the neat or blended application in the transportation sector without the requirement of major technical adaptation [1-3,6].

The lignocellulose-to-bioethanol process consists of five unit operations; a) pre-treatment of the feedstock, b) production of the (hemi-) cellulolytic enzymes, c) enzymatic hydrolysis of the pre-treated feedstock, d) fermentation of the hydrolyzate to bioethanol, and e) down-stream processing [3,6-10]. In the last two decades, all five unit operations have been subjected to intensive research activities, which resulted in key technologies with improved yields and efficiencies. Thus, different biological, chemical, and physical pre-treatment methods have been developed and are applied alone or in combination to increase the enzyme accessibility and to facilitate cellulose depolymerization [7,9,11]. Further process intensification was achieved by combination of two or more process steps into one single unit operation [5,9], most importantly the simultaneous saccharification and (co-) fermentation (SS(C)F) process which has been applied in bioethanol production at laboratory [12,13] and pilot plant [1,14] scale. Genetic and evolutionary engineering enabled *Saccharomyces cerevisiae* to convert both glucose and xylose, the major hemicellulose-derived sugar, and it enhanced the organism’s robustness towards inhibitory compounds (e.g., furans, acids, and phenolic compounds) which are by-products formed during pre-treatment of the feedstock [6,8,10,15-21].

Unit operations within a process are often strongly interlinked so that variation of process parameters in one unit operation can impact the overall process efficiency through indirect effects on other unit operations. Optimization studies, therefore, must implement a complete mass balance-based process analysis and not only focus on single unit operations isolated from the respective others in the process. Despite the extensive research efforts made in R&D facilities in the recent past, there is currently still a lack in comprehensive analyses done at the level of the whole process [1,3]. Besides techno-economic analyses published since the mid-1980s [1,2,4,5,22], data are mainly available from a few pilot scale plants such as the SEKAB plant in Sweden [1,23,24]. The scale of pilot plants, however, already excludes high-throughput analysis of different process configurations. In contrast, data acquired at laboratory scale might not be characteristic and lack transferability for larger scale productions.

**Figure 1:**
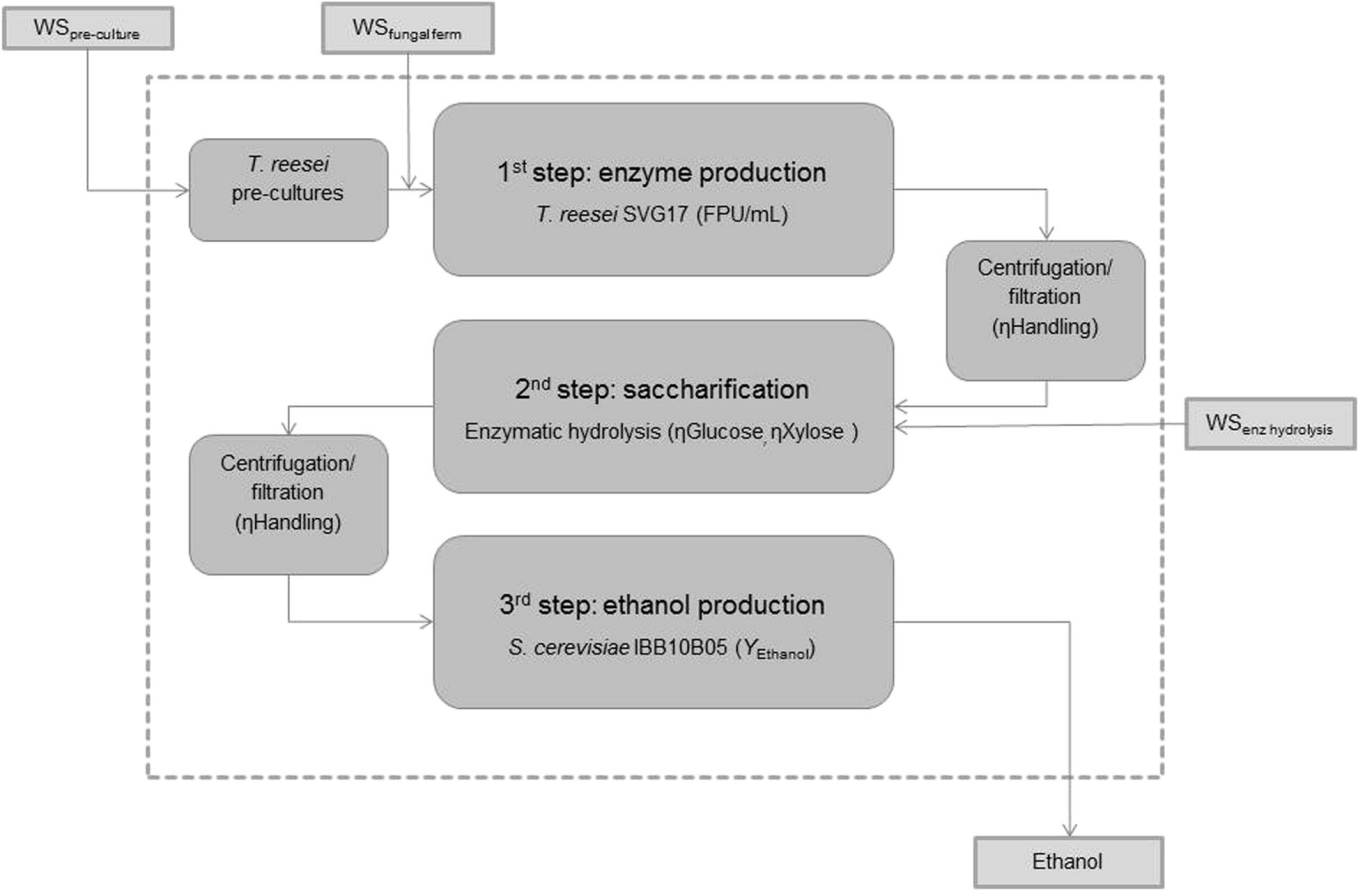
**An overview of the laboratory scale SHCF process.**

In this study, we present an integrative analysis of a lignocellulose-to-bioethanol process on representative laboratory scale (90 mL to 4 L). The process was run as separate hydrolysis and co-fermentation (SHCF), which ensures optimal conditions for both hydrolysis and mixed glucose-xylose fermentation [19,25]. Further, SHCF, as compared to simultaneous saccharification and co-fermentation (SSCF), reduces the complexity of the process, which is important for larger scale applications. Consequently, lignocellulose-to-bioethanol processes which are heading towards industrial scale (e.g., POET-DSM ‘Project Liberty,’ Clariant AG ‘SunLiquid’) are run as SHCF. An overview of the three-step process is given in Figure 1. Firstly, a fraction of the pre-treated wheat straw was used for cultivation of a *Trichoderma reesei* strain SVG17 for the production of (hemi-) cellulolytic enzymes. ‘On-site’ enzyme production is economically advantageous and efficient [26] and it reduces the cost and supply dependency on commercial enzyme suppliers. Despite that, research and pilot scale studies almost exclusively rely on commercially available enzymes [1,2,14,23,26]. The enzyme solution is then utilized for the saccharification of the pre-treated wheat straw (Figure 1). Since fungal cultivation was conducted on the same feedstock, the enzyme mixture is highly optimized for this substrate. The resulting hydrolyzate contains glucose and xylose, and both sugars are converted efficiently to ethanol by the application of the genetically and evolutionary engineered *S. cerevisiae* strain IBB10B05 [19,27].

As shown in Figure 1, the single unit operations were integrated to one SHCF process and mass balance analysis was performed. The resulting overall process ethanol yield (*Y*_Ethanol-Process_), the amount of ethanol produced from 1 kg of dry mass feedstock, thereby quantifies the process efficiency, and it is the key parameter to assess and compare different processes [1,2,14,23]. Consequently, mass balance analysis and *Y*_Ethanol-Process_ were used in this study to investigate different process configurations, and it generated an in-depth knowledge of the SHCF process.

The aim of this study was to establish a tool for process analysis already on laboratory scale. Integration of unit operations and balance-based analysis of process configurations identified bottlenecks and potential optimization targets within the SHCF process. An integrative process analysis at an early stage can render process development more efficient and therefore might contribute to the success of second-generation bioethanol production.

## Results and discussion

### The feedstock

The substrate presented in this study was Austrian wheat straw pre-treated with steam explosion. Wheat straw has a high potential as sustainable biomass source in Europe based on its abundance and low cost [28]. Steam explosion, in combination with chemicals or alone, has been described as an efficient and cost-effective method for pre-treatment of wheat straw [9]. The raw material in this study was treated with a simple method based on steam explosion only. The pre-treated wheat straw had a dry mass (DM) content of approximately 90% and thereof the water insoluble fraction was 69%. The compositional analysis is depicted in Table 1. The acid hydrolyzate contained majorly glucose and xylose. Other hemicellulose-derived sugars (e.g., L-arabinose, galactose) were only present in amounts below the detection limit of the high performance liquid chromatography (HPLC) system and are not mentioned in Table 1. Although processed under the same conditions, the pre-treated wheat straw composition showed batch-to-batch variability. Thus, the glucose and xylose content varied by 5% and 13%, respectively (Table 1). Variation in the pre-treated feedstock composition, especially in xylose content, has been observed before [25]. The amorphous nature of the hemicellulose and different levels of degradation during the pre-treatment as well as seasonal variations are likely explanations. Since both batches of wheat straw were utilized throughout this study, analysis of unit operation and mass balanced process analysis were based on averaged values (Table 1). The raw material before pre-treatment had a xylose content of 24.9 ± 0.4% and a glucose content of 36.1 ± 0.1% dry matter (data not shown). Mass losses caused by pre-treatment were 10% on average.

**Table 1:** **Compositional analysis of the pre-treated wheat straw**

**Components in dry matter**	**Percentage [%]**		
	**Batch 1**	**Batch 2**	**Mean**
Carbohydrates			
Glucose	43.7	48.7	46.2 ± 2.5
Xylose	17.2	13.1	15.2 ± 2.0
Non-carbohydrates			
Acid-soluble lignin	1.3	2.0	1.7 ± 0.3
Acid-insoluble lignin	27.7	30.0	28.9 ± 1.2
Ashes	4.5	4.4	4.5 ± 0.0
Others	5.6	4.3	4.9 ± 0.6

### Experimental analysis of unit operations

The three unit operations of the SHCF process, enzyme production, hydrolysis, and fermentation, were analyzed under varying process conditions. As shown in Figure 1, the process streams between the unit operations were treated by centrifugation, filtration, and concentration. The resulting losses were included into the process analysis with the efficiency factor of conditioning steps (ηHandling), and it was determined to be 75% on average.

#### Production of (hemi-) cellulases by *T. reesei* SVG17

**Figure 2:**
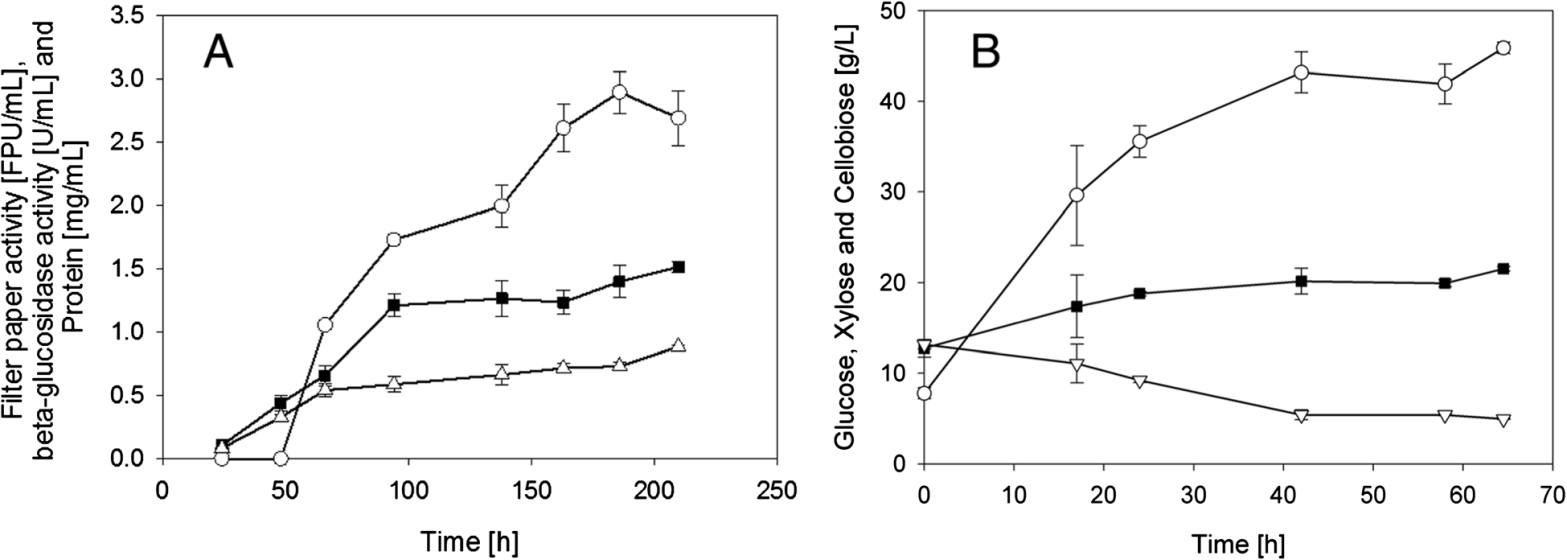
**Time courses of the** ***T. reesei*** **SVG17 fed-batch fermentation (A) and the enzymatic hydrolysis (B).** Thirty grams DM WS was added after 66, 94, and 138 h to the fungal fermentation. Hydrolysis reaction was run with 30 FPU/g_DM WS_. Both time courses represent mean values from two experiments. Symbols: **(A)** Total cellulase activity (empty circles), beta-glucosidase activity (filled squares), and protein concentration (empty triangles). **(B)** Glucose (empty circles), xylose (filled squares), and cellobiose (empty triangles).

*T. reesei* is the majorly applied organism for (hemi-) cellulase production, and it has been studied and continuously improved since the 1960s [29-33]. The herein presented *T. reesei* SVG17 is a mutant of the QM9414 strain, and previous studies have described it as useful enzyme producer at both laboratory and pilot scale [31]. Fungal cultivations were run as batch fermentation with a substrate loading of 30 g DM pre-treated wheat straw per L (g_DM WS_/L). In 7 days of fermentation, a total volumetric cellulolytic activity of 1.7 ± 0.1 FPU/mL was reached. The beta-glucosidase activity was determined to be 0.6 ± 0.1 U/mL. To increase the enzyme yields, fermentations were also run as fed-batch. Per 2 L of fermentation, 30 g_DM WS_ was added three times after 66, 94, and 138 h of fermentation. The time course of the fed-batch fermentation is depicted in Figure 2A. In 210 h of fermentation, the volumetric cellulase activity reached 2.7 ± 0.02 FPU/mL. Similar to the batch fermentation, the beta-glucosidase activity was approximately half of the FPU/mL value and it was determined to be 1.5 ± 0.02 U/mL. Addition of feedstock was described to prolong the phase in which enzyme production is most active by freshly inducing both biomass growth and enzyme expression [30,32]. Consequently, the cellulase activity is increasing after each addition of WS, and only towards the end (180 to 210 h), the FPU/mL-time curve is stagnating. The time courses of beta-glucosidase activity and protein concentration show a similar pattern with a less pronounced effect of the feed. In the fed-batch fermentation, it was possible to improve the volumetric cellulase activity 1.6-fold as compared to the batch fermentation. This increase, however, required a 2.5-fold higher substrate loading. The impact of both process configurations on *Y*_Ethanol-Process_ was evaluated with mass balance analysis and will be discussed hereinafter.

Despite the difficult substrate conditions, *T. reesei* SVG17 was able to grow on the pre-treated wheat straw and showed efficient and reproducible production of (hemi-) cellulolytic enzymes. A drawback of strain SVG17 is the relatively low beta-glucosidase activity in the enzyme solution which did not exceed 50% of the overall cellulase activity (measured in FPU). A beta-glucosidase activity to FPU ratio of 1 was found to be the lower limit for efficient biomass conversion [34,35]. Low beta-glucosidase activity is an often observed problem in the production of cellulolytic enzymes by *T. reesei*. To overcome this problem, genetically engineered strains overexpressing heterologous beta-glucosidase genes have been described in the literature [34-37]. Although the availability of a robust *T. reesei* strain producing a beta-glucosidase-boosted enzyme solution could be important in view of an optimized process output, the aim of this study was not primarily optimization itself, but rather provision of a *basis for optimization* through integrative mass balance analysis of a representative SHCF process. Therefore, the use of *T. reesei* strain SVG17 was fully in line with the concept of the study, and the limitation in beta-glucosidase noted was not considered to restrict the relevance of the current investigation. Moreover, the effects of enhanced beta-glucosidase activity on hydrolysis yield and process performance are described later in this manuscript.

#### Enzymatic hydrolysis

To render lignocellulose-to-bioethanol processes economically feasible, a high ethanol titer is crucial [17]. Consequently, hydrolysis reactions must aim for high solid loadings to increase the sugar content. This, in turn, does increase the content of compounds, which are potentially toxic for the fermentation organism. In a previous study, we have shown that the solid loading in the hydrolysis reaction can be increased from 5% to 15% DM WS without introducing severe inhibition effects on *S. cerevisiae* strain IBB10B05 [19]. Enzyme loadings were varied. Firstly, reactions were run with 25 FPU/g_DM WS_ [19,27] and the final hydrolyzate contained 40.6 ± 5.7 g/L glucose and 18.0 ± 2.3 g/L xylose. Based on the compositional analysis of the feedstock (Table 1), this equals a conversion efficiency of 60% for glucose (ηGlucose) and 81% for xylose (ηXylose). To improve the conversion efficiencies, the enzyme loading was increased to 30 FPU/g_DM WS_, and ηGlucose of 67% and ηXylose of 95% were reached. The 15% hydrolyzate contained 46.1 ± 0.2 g/L glucose and 21.5 ± 0.2 g/L xylose. The time course of the 30 FPU/g_DM WS_ hydrolysis reaction is depicted in Figure 2B. In addition to glucose and xylose, a considerable amount of cellobiose (5.0 g/L, Figure 2) was released into the hydrolyzate. Cellobiose is known to have an inhibitory impact on cellulases (e.g., [38,39]). Accumulation of cellobiose during hydrolysis is caused by limitation in beta-glucosidase activity in the enzyme mixture used. To evaluate the impact of enhanced beta-glucosidase activity, hydrolysis reactions (30 FPU/g_DM WS_) were additionally performed with supplemented Novozyme188. The FPU to beta-glucosidase activity was 1, which was chosen according to the literature [34,35]. The resulting hydrolyzate had a glucose and xylose concentration of 51.8 ± 1.1 g/L and 23.0 ± 1.0 g/L, respectively. The cellobiose concentration was below 1.5 g/L. Addition of beta-glucosidase increased the ηGlucose from 67% to 78%. The already high ηXylose was further increased and reached full conversion.

#### Fermentation to ethanol with *S. cerevisiae* strain IBB10B05

*S. cerevisiae* IBB10B05 proved to be a sturdy and efficient fermentation strain for mixed glucose-xylose fermentation in spent sulfite liquor, wheat straw hydrolyzates, and a combination thereof [19,27]. Strain IBB10B05 performs excellently under most basic process and substrate conditions. Thus, fermentations were run in simple batch cultures without process monitoring and control (e.g., pH adjustment). The 15% hydrolyzate was applied without pre-treatment (e.g., detoxification) and yeast extract was added as a sole substrate supplement. An in-depth physiological characterization of strain IBB10B05 in fermentation of 15% hydrolyzates has been published recently [19]. The fermentation time course is depicted in Additional file 1. Both glucose (~37 g/L) and xylose (~19 g/L) were depleted within 50 h of fermentation, and in total, 22 g/L ethanol was produced within this time frame (Additional file 1, [19]). This represents an ethanol yield of ~0.4 g/g_Glc + Xyl_.

### Integration of unit operations and process analysis

**Figure 3:**
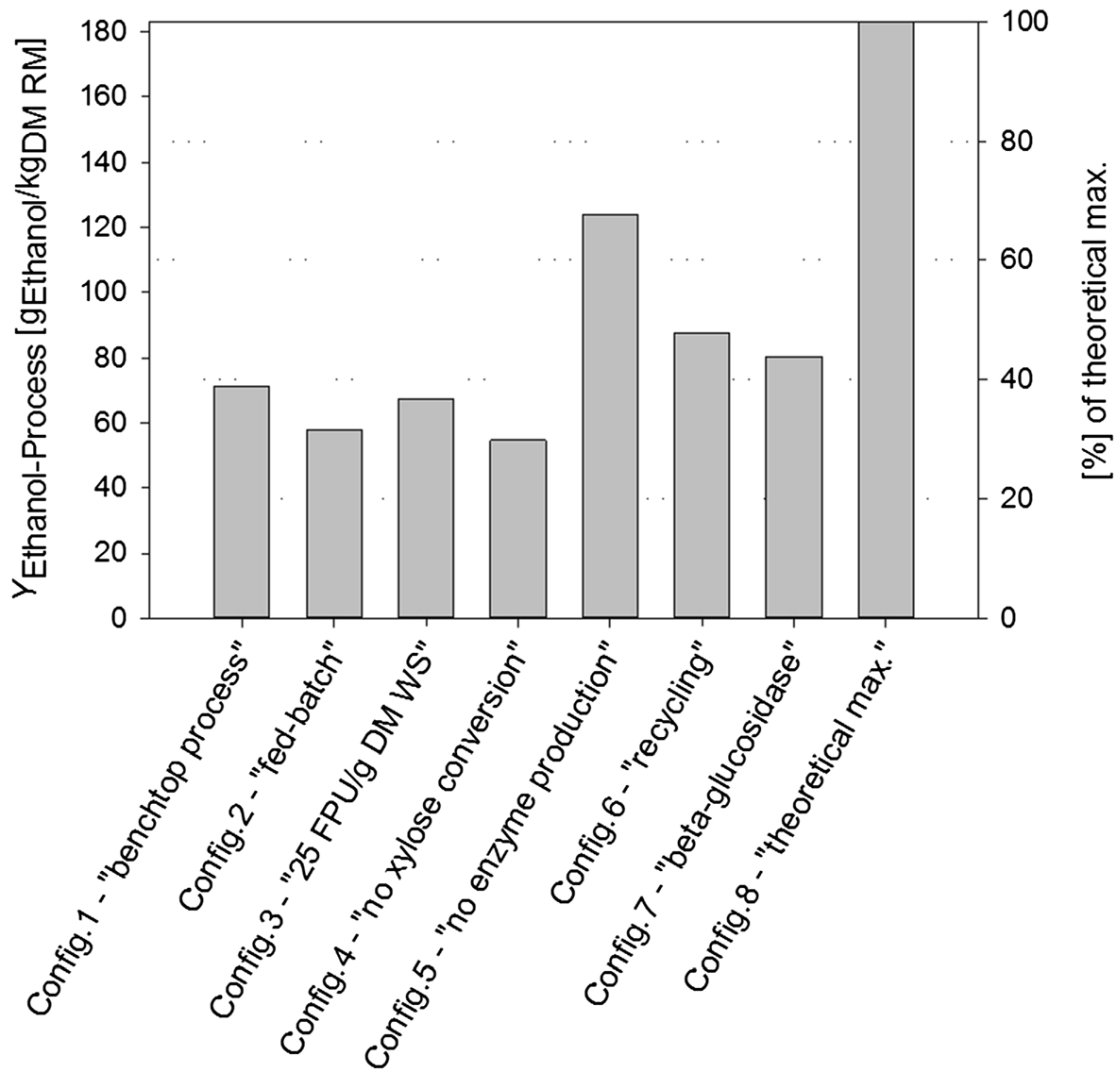
**The influence of different process configurations on** ***Y***_**Ethanol-Process**_ **.** Detailed description of process parameters are summarized in Additional file 2.

The single unit operations, enzyme production, hydrolysis, and fermentation, were integrated to one SHCF process as depicted in Figure 1. To evaluate and compare the different process configurations in context of the complete SHCF process, mass balance analysis was performed. The different process configurations are described based on critical output parameters (FPU/mL, ηGlucose, ηXylose, ηHandling, and ethanol yield (*Y*_Ethanol_)), and the corresponding mass balance analyses are summarized in Additional file 2. A comparison of process configurations based on *Y*_Ethanol-Process_ is depicted in Figure 3. Note that to account for losses caused by pre-treatment and to facilitate comparison of the laboratory scale SHCF process with data from the literature, mass balance analyses were based on the raw material. It was assumed that the required input of raw material was 10% higher as the input calculated for the pre-treated wheat straw (Additional file 2). Throughout this study, *Y*_Ethanol-Process_ is given in g ethanol produced per kg raw material (g_Ethanol_/kg_DM RM_).

Enzyme production, the first unit operation of the SHCF process, was run as batch or as fed-batch. The latter approach resulted in a higher volumetric activity but also required a higher substrate loading (‘Production of (hemi-) cellulases by *T. reesei* SVG17’). When enzymes were produced in batch fermentations (Config.1), the resulting *Y*_Ethanol-Process_ was 71.2 g_Ethanol_/kg_DM RM_. In fed-batch fermentations (Config.2), *Y*_Ethanol-Process_ was 58.0 g_Ethanol_/kg_DM RM_, which is a 19% decrease as compared to Config.1. This clearly emphasizes the need for evaluating process parameters in context of the complete process. Despite the extensive research on cellulase production in *T. reesei* (e.g., [29,32,40,41]), the influence of substrate or process conditions on the success of the overall bioethanol production process is scarcely considered. Enzymatic hydrolysis, the second unit operation, was analyzed with two different enzyme loadings, 25 FPU/g_DM WS_ (Config.3) and 30 FPU/g_DM WS_ (Config.1). Config.3 resulted in an overall process ethanol yield of 67.5 g_Ethanol_/kg_DM RM_ (Additional file 2, Figure 3). This is 5% less as compared to the reaction with 30 FPU/g_DM WS_ (*Y*_Ethanol-Process_ 71.2 g_Ethanol_/kg_DM RM_). The last unit operation, the fermentation to ethanol, was accomplished with the xylose-fermenting *S. cerevisiae* strain IBB10B05 (Figure 1). To compare the efficiencies of a SHCF and a separate hydrolysis and fermentation (SHF) process, mass balance analysis was additionally performed with glucose conversion only (Config.4). With an ethanol on glucose yield of 0.45 g/g_Glc_ [19,27], the *Y*_Ethanol-Process_ was 54.6 g_Ethanol_/kg_DM RM_ (Additional file 2, Figure 3). This is 23% less as compared to Config.1. Although, genetically engineered xylose-fermenting *S. cerevisiae* strains have been described extensively in the literature (e.g., [10,15]), pilot plants often still operate with non-GM yeasts and rely on glucose conversion only [1,23]. This integrative process analysis, however, clearly highlights the importance of efficient conversion of all sugars in the hydrolyzates and strain IBB10B05 proved to be an excellent candidate.

Integration of unit operations and analyses of the different process configurations (Config.1 to 3) showed that Config.1 has the highest *Y*_Ethanol-Process_ (Figure 3) and the mass balance analysis is depicted in Table 2. Thus, enzyme production in batch fermentation (working volume 4 L) and processing of the enzyme solution (ηHandling 75%) resulted in a total of 5,100 FPU. With an enzyme loading of 30 FPU/g_DM WS_, it was possible to hydrolyze 170 g DM WS. After treatment of the 15% hydrolyzate (ηHandling 75%), 39.5 g glucose and 18.4 g xylose were available for fermentation, which was converted to 23.1 g of ethanol. A total process ethanol yield of 71.2 g_Ethanol_/kg_DM RM_ was reached (Table 2).

**Table 2:** **Mass balance analysis of the benchtop SHCF process**

	**Input**	**Output**
**1st step: enzyme production (** ***T. reesei*** **SVG17)**		
Pre-cultures (WS_pre-culture_)	5.6 g DM WS	
Batch cultivation (WS_fungal ferm_)	120 g DM WS	
Total cellulolytic activity		1.7 FPU/mL
ηHandling 75%		5,100 FPU total
**2nd step: saccharification (enzymatic hydrolysis)**		
Substrate loading (WS_enz hydrolysis_)	170 g DM WS	
Glucose (ηGlucose 67%, ηHandling 75%)		39.5 g
Xylose (ηXylose 95%, ηHandling 75%)		18.4 g
**3rd step: ethanol production (** ***S. cerevisiae*** **IBB10B05)**		
Ethanol (*Y*_Ethanol_ 0.4 g/g_Glc+Xyl_)		23.1 g
**Total**		
Substrate loading - pre-treated	295.6 g DM WS	
Substrate loading - raw material	325.2 g DM RM	
Ethanol		23.1 g
*Y*_Ethanol-Process_	71.2 g_Ethanol_/kg_DM RM_	

An overview of the process is depicted in Figure 1. Boundary conditions: batch fermentations with 30 g_DM WS_/L, 15% DM WS, and 30 FPU/g_DM WS._

To assess the efficiency, the benchtop SHCF process was compared to currently available data from pilot (SEKAB, IBUS, BCyL) and commercial (Clariant, ‘SunLiquid’) scale plants. A summary of process configurations, plant capacities, and *Y*_Ethanol-Process_ is depicted in Table 3. The pilot scale plants summarized in Table 3 operate without ‘on-site’ production of (hemi-) cellulolytic enzymes. To still render a comparison of the process efficiencies possible, mass balance analysis of the laboratory scale SHCF process was performed excluding enzyme production (Config.5, Figure 3 and Additional file 2). Without the loss of feedstock required for the fungal cultivation, the *Y*_Ethanol-Process_ was 123.7 g_Ethanol_/kg_DM RM_, which is 1.7-fold higher as compared to Config.1. This is already within the range of *Y*_Ethanol-Process_ reported for the pilot scale plants (118 to 157.8 3 g_Ethanol_/kg_DM RM_; Table 3), clearly highlighting the usefulness of the herein presented laboratory scale SHCF process as a model for establishing an integrative process analysis. However, direct comparison of Config.1 and Config.5, solely based on *Y*_Ethanol-Process_, is not sufficient. ‘On-site’ enzyme production has been described to entail several advantages, such as being cost-effective and efficient [26,42]. Thus, for further evaluation of the feasibility of the process configurations, a detailed economic analysis, as published for other process [2,14,22], must be conducted.

**Table 3:** ***Y***_**Ethanol-Process**_ **for commercial, pilot, and laboratory scale processes**

**Substrate**	**Capacity [1,000 t**_**DM RM**_ **/year]**	**Process**	***Y***_**Ethanol-Process**_ **[kg**_**Ethanol**_ **/t**_**DM RM**_ **]**	**Reference**
Cereal straw	150	SHCF + E^a^	222.2	Clariant ‘SunLiquid’ [42]
Wheat straw	8.8	SSF	123	‘IBUS’ [23]
Forestry residues	0.7	SSF	118 to 157.8	‘SEKAB’ [1]
Wheat straw	25.6	SHF	154	‘BCyL’ [1]
Wheat straw	-	SHCF/+E^a^	123.7/71.2	This study

^a^‘On-site’ enzyme production.

The by far most efficient process is the ‘SunLiquid’ process, which is operated as SHCF with implemented ‘on-site’ enzyme production. The reported *Y*_Ethanol-Process_ is 222.2 g_Ethanol_/kg_DM RM_ (Table 3) [42]. This is 18% higher as compared to the maximum theoretical yield of the benchtop SHCF process which was determined to be 183.1 g_Ethanol_/kg_DM RM_ (Config.8, Figure 3 and Additional file 2). There are several factors that could explain the exceptionally high *Y*_Ethanol-Process_ described for the ‘SunLiquid’ process. The first factor is the pre-treatment method applied. Ideally, pre-treatment of lignocellulosic biomass enriches the structural carbohydrate cellulose and hemicellulose by removal of the lignin and enhances the accessibility of the partially crystalline cellulose. It therefore has an impact on the efficiency of the fungal fermentation and the enzymatic hydrolysis and is directly affecting *Y*_Ethanol-Process_ [7,11]. In this study, the steam explosion was performed as batch and running the process continuously could reduce mass losses caused by pre-treatment. However, evaluation of varying pre-treatment methods was beyond the scope of this study and is not considered in more detail hereinafter. The second factor influencing *Y*_Ethanol-Process_ is the potential application of enzyme or solid recycling to boost the hydrolysis efficiency [43,44]. This process option was also analyzed in context of this study. With an estimated increase in glucose released per enzyme loading of 35% (e.g., [43,44]), the *Y*_Ethanol-Process_ of the benchtop SHCF process would improve to 87.8 g_Ethanol_/kg_DM RM_ (Config.6, Figure 3 and Additional file 2). The third factor is the organism employed for enzyme production. The importance of a *T. reesei* strain secreting a balanced enzyme mixture has been described in the literature [34-37]. In this study, the application of an enzyme solution with a beta-glucosidase to FPU ratio of 1 was investigated. The resulting *Y*_Ethanol-Process_ was 80.3 g_Ethanol_/kg_DM RM_ (Config.7, Figure 3 and Additional file 2). In comparison to Config.1, the altered process configurations resulted in an increase in *Y*_Ethanol-Process_ of 19% (Config.6) and 11% (Config.7). However, the process yields did not exceed 50% of the reported *Y*_Ethanol-Process_ of the ‘SunLiquid’ process. Therefore, we took the process analysis one step further and analyzed the benchtop SHCF process towards potential bottlenecks.

**Figure 4:**
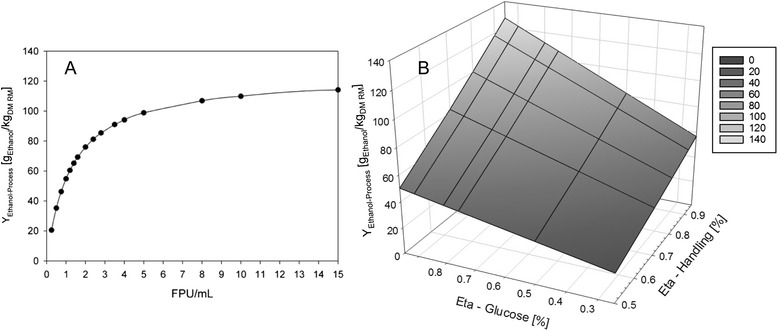
**The influence of key parameters on** ***Y***_**Ethanol-Process**_ **. (A)** *Y*_Ethanol-Process_ in dependence of the total cellulolytic activities produced by fungal fermentation (boundary conditions: batch with 30 g_DM WS_/L, 30 FPU/g_DM WS,_ ηGlucose 67%, ηXylose 95%, ηHandling 75%, and *Y*_Ethanol_ 0.4 g/g_Glc+Xyl_). **(B)** Influence of ηGlucose and ηHandling on *Y*_Ethanol-Process_ (boundary conditions: batch with 30 g_DM WS_/L, 1.7 FPU/mL, 30 FPU/g_DM WS_, ηXylose 95%, and *Y*_Ethanol_ 0.4 g/g_Glc+Xyl_).

Based on the detailed mass balance analysis of the integrated benchtop SHCF process and the resulting understanding of the process streams, it was possible to identify three key parameters which exhibit a significant influence on *Y*_Ethanol-Process_, the enzyme yield, ηGlucose, and ηHandling (Figure 1). The correlations between *Y*_Ethanol-Process_ and these parameters are depicted in Figure 4. Firstly, *Y*_Ethanol-Process_ was plotted over the enzyme yield (FPU/mL) (Figure 4A). To increase the *Y*_Ethanol-Process_ of Config.1 to 100 g_Ethanol_/kg_DM RM_, for an instance, a 3.1-fold increase in total cellulolytic activity is required (from 1.7 to 5.3 FPU/mL, Figure 4A). The importance of an efficient enzyme production is further highlighted, by comparing the maximum theoretic *Y*_Ethanol-Process_ observed in this study with the ‘SunLiquid’ process. To enhance the *Y*_Ethanol-Process_ of Config.8 to 222 g_Ethanol_/kg_DM RM_ [42], the enzyme yield must be increased twofold, from 1.7 FPU/mL to 3.4 FPU/mL. In addition to the above discussed factors, a higher enzyme yield during fungal fermentation might therefore explain for the high *Y*_Ethanol-Process_ of the ‘SunLiquid’ process.

As shown in Figure 4B, the overall process efficiency is further influenced by the two parameters ηGlucose and ηHandling. Thus, *Y*_Ethanol-Process_ can vary between 22.4 (ηGlucose 25% and ηHandling 50%) and 127.2 g_Ethanol_/kg_DM RM_ (ηGlucose 95% and ηHandling 95%) under else same boundary conditions. Possible improvement of ηGlucose has already been described within this study. Data summarized in Table 3 suggest that *Y*_Ethanol-Process_ increases with the scale of the plant (‘Capacity’). A correlation between plant capacity and overall yield is further supported by the literature [1-5,14,22-24], and it is suggested that processes are becoming more efficient with increasing scale [1-3,5]. Explicit information on ηHandling for pilot and commercial scale plants are not given. The fact that *Y*_Ethanol-Process_ of Config.5 (“no enzyme production”) is within the range of to the pilot scale plants (Table 3), however, might indicate that the ηHandling are similar. To further increase *Y*_Ethanol-Process_, improvement of ηHandling will be a target of future optimization studies of the benchtop SHCF process.

## Conclusions

In this study, an integrative process analysis of a benchtop SHCF is presented. Based on mass balance analysis, the influence of varying process configurations on *Y*_Ethanol-Process_ was analyzed. Thereby, a fundamental understanding of the complete process was established. This allowed for identification of the process parameters, which have the highest impact on *Y*_Ethanol-Process_, the enzyme yield, ηGlucose, and ηHandling. It was further shown that, under comparable process conditions (Conifg.5 - no enzyme production), *Y*_Ethanol-Process_ of the benchtop SHCF process is equal to pilot scale plants. We therefore believe that the benchtop-scale analysis described herein presents an important and useful tool to identify bottlenecks and optimization targets within the process with reasonable effort and expenditure. Findings of this study stress the importance to establish a mass balance-based understanding of the lignocellulose-to-bioethanol process and to optimize unit operations or process parameters with respect to *Y*_Ethanol-Process_.

## Methods

### Chemicals and media used

Unless mentioned differently, all chemicals were from Carl Roth + Co KG (Karlsruhe, Germany). Mineral (M-) media for cultivation of *T. reesei* SVG17 contained 5 g/L yeast extract, 5 g/L KH_2_PO_4_, 3.75 g/L (NH_4_)_2_SO_4_, 0.3 g/L MgSO_4_ × 7H_2_O, 0.3 g/L CaCl_2_ × 2H_2_O, and 1 mL/L trace elements (5 g/L FeSO_4_ × 7H_2_O, 1.6 g/L MnSO_4_ × H_2_O, 1.4 g/L ZnSO_4_ × 7H_2_O, 0.2 g/L CoCl_2_ × 6H_2_O, 15 g/L EDTA disodium chloride salt). Additionally, 0.2 mL/L Tween 80 (Sigma-Aldrich, St. Louis, MO, USA) and 0.5 g/L rapeseed oil were supplied. Potato-dextrose-agar (PDA) was prepared as described by the manufacturer. Yeast extract peptone glucose (YPD) medium for cultivation of *S. cerevisiae* contained 10 g/L yeast extract, 20 g/L peptone from casein, and 20 g/L glucose. In 15% hydrolyzate conversion experiments, 10 g/L of yeast extract was added as a sole medium supplement.

### Preparation of the lignocellulosic feedstock

Pre-treated wheat straw from Austria was kindly provided by the University of Applied Sciences in Upper Austria (FH Wels). Throughout this study, two batches of feedstock were received and they were both pre-treated as described in the following. The wheat straw was air-dried to a water content of approximately 8% (*w*/*w*), and the fibers were chaffed to reduce the length of the fibers to below 8 cm. Further, the wheat straw was treated by steam explosion at 200°C, 15 bar for 10 min, with a water to wheat straw ratio of 1. After cooling, the wheat straw was dried to a dry mass content of 90% and stored at 4°C in plastic bags. Per batch, DM and water insoluble (WIS) content was analyzed in triplets. For the former, a moisture analyzer (MA 50, Sartorius AG, Göttingen, Germany) operated at 105°C was used. For determination of the WIS content, 2 g of wheat straw was washed with 50 mL of 50°C warm water, dried at 105°C for 24 h, and weighted. The content of structural carbohydrates, lignin, and ash in the wheat straw was analyzed following the protocol of the National Renewable Energy Laboratory (NREL) [45].

### Strains

For the production of the (hemi-) cellulolytic enzymes, the *T. reesei* strain SVG17 was applied. The strain background has been described in a previously published study [31]. For conversion of the 15% hydrolyzate to ethanol, the *S. cerevisiae* strain IBB10B05 was utilized. It is a descendant of the BP10001 strain, which was further improved by evolutionary engineering. Detailed descriptions of the construction of the BP10001 strain and the evolution strategy have been published elsewhere [18,20].

### Bioreactor cultivations for the production of the (hemi-) cellulolytic enzymes

*T. reesei* SVG17 was maintained on PDA plates at 4°C. The fungus was revitalized by transfer of a piece of overgrown agar onto fresh PDA plates. Incubation was for 3 days at 30°C. Starter cultures were prepared in 300-mL wide mouth shake flasks without baffles filled with 200 mL of mineral medium (M-medium) containing 14 g_DM WS_/L. Fermentation was started by transfer of a piece of overgrown PDA agar to the fermentation media. Incubation was for 5 days at 30°C, 200 rpm, and pH 4.5 in a Certomat BS-1 orbital incubator shaker (Sartorius AG, Göttingen, Germany). Subsequently pre-cultures were pooled to ensure homogeneous biomass composition.

Main cultivations were run in batch or fed-batch mode in benchtop bioreactors (Labfors III, Infors AG, Bottmingen, Switzerland) with 2 L as working volume. The bioreactors are equipped with two six-plated impellers. The reactor diameter to impeller diameter ratio was 3 and the reactor height to reactor diameter ratio was 1.5. Cultivations were accomplished in duplicates (two parallel bioreactors). M-media was supplemented with 30 g_DM WS_/L, and the fermentation was started by transfer of 200 mL of the pooled pre-cultures into the bioreactor. Fermentation conditions were as follows: 30°C, pH 4.5 (adjusted online with 1 M KOH and 3%, by volume NH_3_), and a dissolved oxygen concentration of 20%. The latter was adjusted continuously with an agitation (200 to 800 rpm) and aeration (0.16 to 1.5 L/min pressurized air) cascade. Incubation was for 7 days. In fed-batch mode, 30 g DM WS and media supplements for 1 L of fermentation media was added per bioreactor after 66, 94, and 138 h of fermentation. Fed-batch experiments were prolonged to 9 days of fermentation time. During the fermentation, samples were frequently removed and centrifuged (15,700*g*, 4°C for 10 min, Eppendorf 5415 R, Eppendorf, Hamburg, Germany) and the supernatant stored at 4°C for analysis. Enzyme activity was evaluated in beta-glucosidase and total cellulolytic activity (given in filter paper units, FPU/mL). FPU activity was measured following International Union of Pure and Applied Chemistry (IUPAC) recommendations [46]. Beta-glucosidase activity was determined as described previously [47], with the following alterations. Two hundred fifty microliters of diluted enzyme solution was mixed with 250 μL of 2.0 mM para-nitrophenyl-β-D-glucopyranoside solution (in 50 mM sodium acetate buffer, pH 5.0) and incubated at 50°C for 10 min. The reaction was stopped with 1 M Na_2_CO_3_. Protein concentration was determined with the Bradford method [48]. Proteins were precipitated and quantified utilizing the Roti-Quant kit (Roth) and following the manufacturer’s instructions.

The enzyme solution was harvested and prepared for the subsequent enzymatic hydrolysis as described in the following. The supernatant of the fungal cultivation was collected by centrifugation (4,420*g*, 4°C; 20 min; Sorvall RC-5B; Thermo Fisher Scientific Inc., Waltham, MA, USA), concentrated by evaporation (45°C, 40 mbar, Laborota 4000, Heidolph Instruments GmbH & Co. KG, Schwabach, Germany) to one tenth of its original volume, filtrated sterile (Whatman Klari-Flex System; GE Healthcare, Little Chalfont, UK), and stored at 4°C. The loss of enzyme solution was included into the mass balance analysis with the efficiency factor ηHandling (Figure 1) and was determined by measuring volume and mass before and after the processing steps.

### Enzymatic hydrolysis of the wheat straw

The substrate loading for the hydrolysis reaction was 15%, by weight dry mass wheat straw. Two different enzyme loadings were applied, 25 and 30 FPU/g_DM WS_. Additionally, one hydrolysis reaction (30 FPU/g_DM WS_) was performed with supplemented beta-glucosidase (Novozyme188; Sigma-Aldrich) to a total activity of 30 U/g_DM WS_. Reactions were performed in 10 mM sodium acetate buffer (pH 4.8) in 500-mL shake flaks with ground-in glass stoppers. Total mass of the reaction was 260 g. The wheat straw suspension was autoclaved and the enzyme added aseptically. Incubation was 50°C, 200 rpm for 72 h (Certomat BS-1). During the hydrolysis, samples were taken. Immediate sample work-up included the boiling of the reaction mixture (100°C, 15 min. 300 rpm, Thermomixer ‘comfort’ Eppendorf) and centrifugation and storage of the supernatant at −20°C for HPLC analysis.

The hydrolyzate was prepared for the subsequent conversion to bioethanol as described in the following. Firstly, it was heated to 100°C for 15 min in a water bath. Remaining solids were removed by centrifugation (4,420*g*, 4°C, 10 min, Sorvall RC-5B) and discarded. The pH of the supernatant was adjusted to 6.5 with 1 M NaOH, and it was filtrated sterile (‘Klari-Flex’) and stored at 4°C. Mass and volume loss was determined before and after the processing of the hydrolyzate and included into the mass balance analysis as ηHandling.

### Shaken-bottle cultivation for bioethanol fermentation

Fermentation of 15% hydrolyzate to ethanol utilizing the *S. cerevisiae* strain IBB10B05 was accomplished as described previously [19]. In brief, seed and starter cultures were prepared aerobically in shaken flask cultures in YPD media at 30°C. Main cultivations were performed anaerobically at 30°C in glass bottles (working volume 90 mL) tightly sealed with rubber septa. Eighty percent of the total volume was wheat straw hydrolyzate, and the remainder volume was composed of 10% yeast extract and 10% inoculum. The starting optical density at 600 nm (OD_600_) was 5. Incubation was at 30°C and 180 rpm (Certomat BS-1) for 7 days. During the fermentation, samples (1.5 mL) were frequently removed from the fermentation media and centrifuged (10 min, 15,700*g*, 4°C, Eppendorf 5415R) and the supernatant stored at −20°C for HPLC analysis.

### HPLC analysis of sugars and metabolites

Sugars (glucose, xylose, arabinose, galactose, and cellobiose) as well as extracellular metabolites (ethanol, glycerol, xylitol, and acetate) were analyzed by HPLC (Merck-Hitachi LaChrome system equipped with an L-7250 autosampler, a Merck L-7490 RI detector, and a Merck L-7400 UV detector). The system was equipped with an Aminex HPX-87H column and an Aminex Cation H guard column (both Bio-Rad Laboratories, Hercules, CA, USA). The operation temperature was 65°C for both columns and the flow rate of the mobile phase (5 mM sulfuric acid) was 0.6 mL/min. The hemicellulose-derived sugars (galactose and arabinose) were only present in minor amounts (<0.5 g/L) and are therefore not further mentioned.

## Additional files

Additional file 1:
**Mixed glucose-xylose fermentation in 15% hydrolyzate utilizing** ***S. cerevisiae*** **strain IBB10B05.** Time course was derived from a previous publication [19]. Symbols: glucose (crosses), xylose (full diamonds), glycerol (empty triangles), xylitol (empty squares), ethanol (empty circles). Additional file 2:
**Summary of in- and output parameters and mass balance analyses of the different process configurations.**

## Abbreviations

SHF: separate hydrolysis and fermentation
SSF: simultaneous saccharification and fermentation
SHCF: separate hydrolysis and co-fermentation
SSCF: simultaneous saccharification and co-fermentation
DM: dry mass
WS: pre-treated wheat straw
RM: raw material
FPU: filter paper unit
Glc: glucose
Xyl: xylose
HPLC: high performance liquid chromatography
IUPAC: International Union of Pure and Applied Chemistry
M-medium: mineral medium
NREL: National Renewable Energy Laboratory
OD_600_: optical density at 600 nm
WIS: water-insoluble
*Y*_Ethanol_: ethanol yield
*Y*_Ethanol-Process_: overall process ethanol yield
YPD: yeast extract peptone glucose
ηHandling: efficiency factor of conditioning steps
ηGlucose/Xylose: conversion efficiency of enzymatic hydrolysis
