# Supplementary figures and images for: From wheat straw to bioethanol: integrative analysis of a separate hydrolysis and co-fermentation process with implemented enzyme production
Source: Biotechnol Biofuels. 2015 Mar 18;8:46. doi: 10.1186/s13068-015-0232-0 (PMC4399083; doi:10.1186/s13068-015-0232-0)

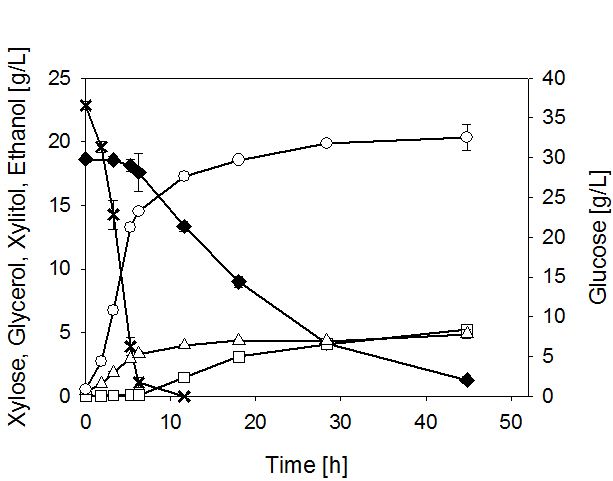

Supplement: Additional file 1: — Mixed glucose-xylose fermentation in 15% hydrolyzate utilizing S. cerevisiae strain IBB10B05. Time course was derived from a previous publication [19]. Symbols: glucose (crosses), xylose (full diamonds), glycerol (empty triangles), xylitol (empty squares), ethanol (empty circles). [file 13068_2015_232_MOESM1_ESM.jpg]
